# Dementia Caregiving and Cognition: An Extension of the Stress Process Model

**DOI:** 10.1093/geroni/igaa057.1542

**Published:** 2020-12-16

**Authors:** Francesca Falzarano, Karen Siedlecki

**Affiliations:** 1 Weill Cornell Medicine, New York, New York, United States; 2 Fordham University, New York, New York, United States

## Abstract

As cases of Alzheimer’s disease and related dementias (ADRD) continue to rise, informal caregivers are critical resources in providing dementia care, yet caregiving is associated with high levels of burden, stress, anxiety and depression. Caregiving can be a prolonged and stressful experience, and impaired cognitive functioning in caregivers could impact their own health and quality of life and compromise the quality of care provided to their care-recipient. Thus, the purpose of the current study is to use the Stress Process Model as a guiding theoretical framework to identify whether primary stressors (e.g., care recipient functional status, cognitive problems) or secondary stressors (e.g., loss of self, economic conflict) predict performance across seven domains of cognition in 50 primary ADRD caregivers. Hierarchical regression analyses were used to examine which primary and secondary stressors emerge as predictors of cognitive performance. Results indicated that primary stressors (e.g., problematic dementia behaviors and relational deprivation) significantly predicted working memory performance and secondary stressors (e.g., economic strain, loss of self) significantly predicted implicit memory performance. Additionally, higher levels of caregiver burden predicted worse performance on executive functioning and implicit memory measures. Overall, the findings of this study indicate that the stress associated with caregiving may have adverse effects beyond psychosocial outcomes, and findings can be used to inform policies and practices with regard to caregiver health and well-being.

